# Ménétrier’s disease in childhood: a case report from China

**DOI:** 10.1186/s12887-020-2005-6

**Published:** 2020-03-06

**Authors:** Jiewei Zhang, Yizhong Wang, Haifeng Liu, Yongmei Xiao, Ting Zhang

**Affiliations:** Department of Gastroenterology, Hepatology, and Nutrition, Shanghai Children’s Hospital, Shanghai Jiao Tong University, 355 Luding Road, Shanghai, 200062 China

**Keywords:** Ménétrier’s disease, Child, Hypertrophy, Hypoalbuminemia

## Abstract

**Background:**

Ménétrier’s disease (MD) is a protein-losing gastropathy characterized by gastric hypertrophy, foveolar hyperplasia and hypoalbuminemia. MD is uncommon in childhood with nonspecific clinical symptoms, and the exact cause of pediatric MD is still unclear.

**Case presentation:**

Here, we reported a 4 year and 10-month boy presenting with MD from China. The patient was suffered with vomiting, abdominal pain, hypoproteinemia and edema. Laboratory tests showed that the boy was infected with *Clostridium difficile* (CD). Gastrointestinal endoscopy revealed giant gastric folds, and histological gastric biopsies showed foveolar hyperplasia with glandular atrophy, infiltration of eosinophils in the lamina propria of the patient. Finally, the boy was recovered after supportive therapy with intravenous albumin and CD eradication.

**Conclusion:**

For the nonspecific clinical symptoms of MD, gastrointestinal endoscopic evaluations with gastric tissue biopsies are required to establish the diagnosis of MD in children with unexplained hypoalbuminemia.

## Background

Ménétrier’s disease (MD) is a rare form of acquired gastropathy that characterized by gastric hypertrophy and hypoalbuminemia, which was first described in 1888 by French pathologist Pierre Ménétrier [1, 2]. The common clinical symptoms of MD include epigastric pain, anorexia, weight loss, nausea, gastrointestinal bleeding, diarrhea, vomiting, fatigue, and peripheral edema [3]. Blood tests of patients with MD show hypoproteinemia and hypoalbuminemia, endoscopy usually reveals giant gastric mucosal folds, and gastric biopsy shows foveolar hyperplasia and decreased oxyntic glands [3]. The etiology of MD is still unknown, but has been associated with some gastric diseases, including gastric bacterial and viral infections [4]. MD can occur both in adults and children. In adults, MD usually presents with an insidious onset and a progressive clinical course, which are related to surgical resection and potential risk for malignant transformation [5–7]. Pediatric MD is generally characterized by abrupt onset and spontaneous regression without any special treatment [8–10]. However, uncommon case of non self-limited pediatric MD needing specific treatment has been reported in previous study [11]. Up to date, limited number of pediatric MD cases was reported in the literature. Here, we report a pediatric case of MD from China. The clinical features of the MD patient were described in the study.

## Case presentation

**Fig. 1:**
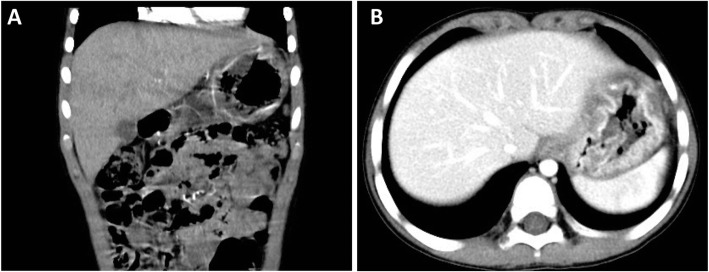
Abdominal computed tomography (CT) images obtained with intravenous Ominpaque showing giant cerebriform enlargement of rugal folds in the gastric fundus and body

**Fig. 2:**
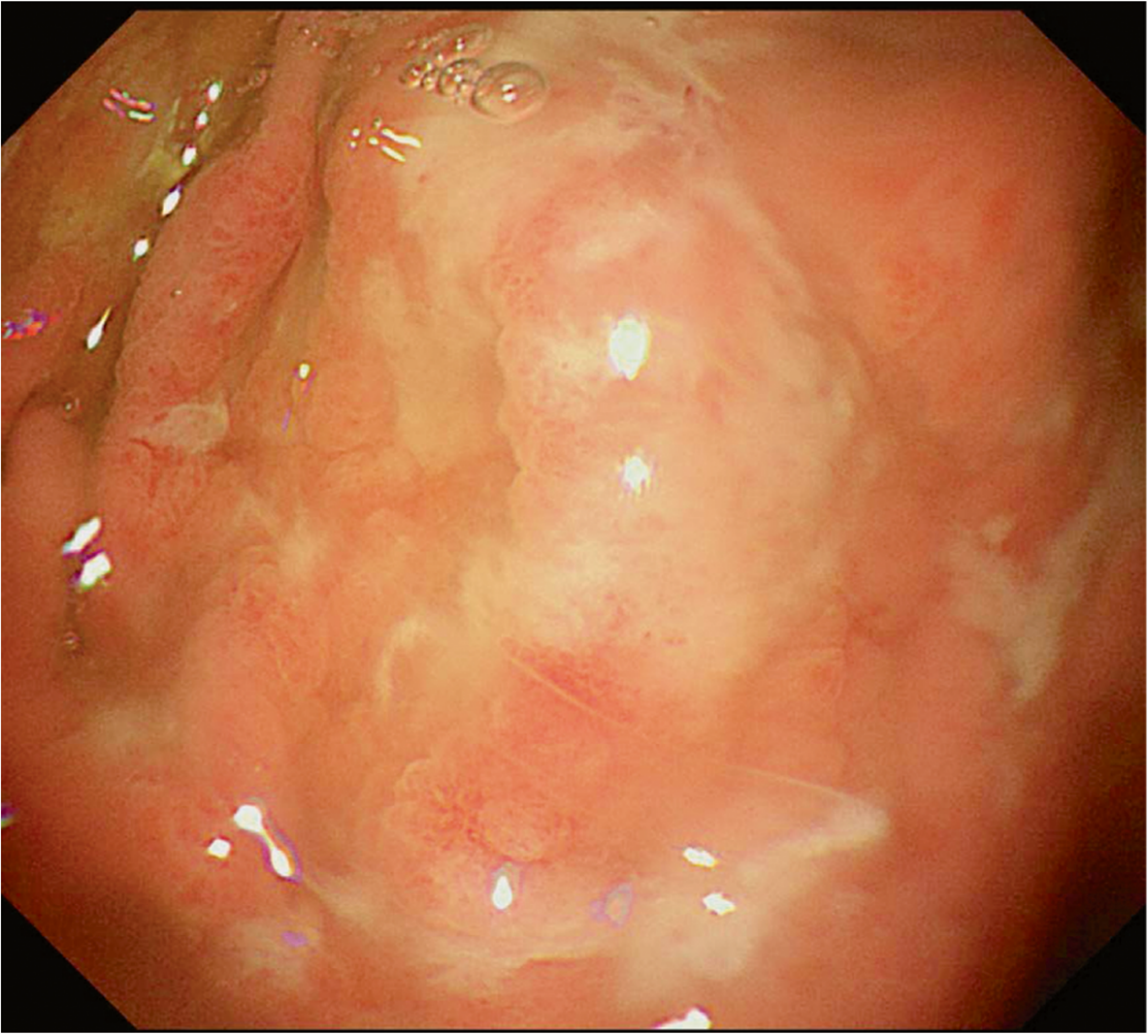
Gastrointestinal endoscopy images showing enlarged gastric rugal folds, erythema, and hemorrhagic erosions in the gastric body

**Fig. 3:**
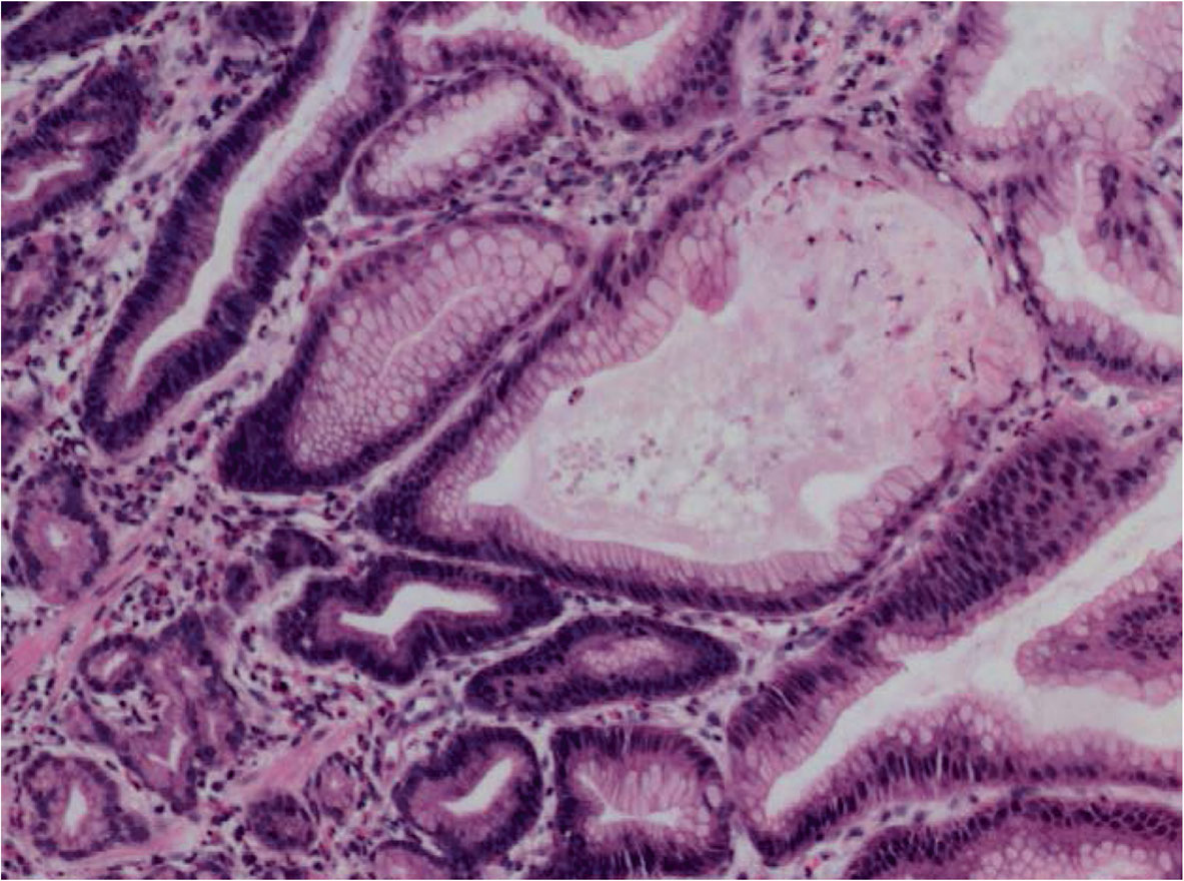
Histological images (H&E, × 100) showing foveolar hyperplasia with glandular atrophy, infiltration of eosinophils, plasmocytes and neutrophils in the lamina propria

The patient was a 4 year and 10-month old boy presented to gastroenterology department of our hospital because of 4 days abdominal pain and vomiting, and 1 day eyelid edema. The boy was born at term with unremarkable family history. Two weeks before the onset of abdominal pain and vomiting, the boy was suffered a course of severe pneumonia caused by *Mycoplasma pneumoniae*. A regimen of ceftriaxone, azithromycin, amoxicillin and potassium clavulanate were given to eradicate the *Mycoplasma pneumonia* infection in respiratory department of our hospital. On admission, physical examination revealed obvious bilateral periorbital edema, abdominal pain around umbilical cord, mild edema of both lower extremities. Laboratory tests showed low levels of total protein (32.99 g/L, reference range: 60-80 g/L), albumin (24.82 g/L, reference range: 38-54 g/L), and globulin (14 g/L, reference range: 22-34 g/L). Hemoglobin level was normal, reticulocyte was slightly up-regulated, and eosinophil percentage was increased (11%). Coagulation function, erythrocyte sedimentation rate were normal, tumor markers (alpha-fetoprotein, carcinoembryonic antigen) and autoantibodies were negative. Parasites antigens, CMV-DNA, and EBV-DNA were all negative. The ^13^C urea breath test was negative. *Clostridium difficile* (CD) toxin test and culture were positive. Serological tests revealed decreased levels of IgG (1.42 g/L, reference range: 3.82–14.04 g/L), complement component 1q (67.27 mg/L, reference range: 159-233 mg/L), C3 (0.43 g/L, reference range: 0.79–1.52 g/L), and C4 (0.08 g/L, reference range: 0.1–0.4 g/L). Abdominal ultrasonography suggested diffuse thickening of the gastric wall. CT showed giant cerebriform enlargement of rugal folds in the gastric fundus and body (Fig. 1), and minimal effusion in the pelvic cavity. Gastrointestinal endoscopy revealed enlarged gastric folds, erythema, and hemorrhagic erosions covered with whitish mucus throughout the gastric body (Fig. 2). Histological findings of the gastric mucosa showed foveolar hyperplasia with glandular atrophy, infiltration of eosinophils, plasmocytes and neutrophils in the lamina propria (Fig. 3). HP was not detected in the gastric biopsies. Combined with the laboratory examination and gastrointestinal endoscopy findings, the patient was diagnosed as giant hypertrophic gastritis (MD), CD infection and hypoproteinemia. The patient was administered with intravenous albumin for six consecutive days (a total of 110 g). A 2-week regimen of oral vancomycin was given to eradicate CD infection. The patient was discharged at the 16th day of admission without notable symptoms, the albumin level was normal (43 g/L) and CD toxin and culture tests were negative. After 2 months of discharge, follow-up gastrointestinal endoscopy showed a mild superficial gastritis, no manifestation of gastric fold hypertrophy and no ulcer. The boy did not complain of any notable symptoms during 6-month of follow-up.

## Discussion and conclusions

MD is a rare disease characterized by hypertrophic folds in the body of the stomach, foveolar hyperplasia and hypoproteinemia due to selective loss of serum proteins across the gastric mucosa [7]. Although MD can occur both in adults and children, pediatric MD presents a distinct clinical course that differs with MD in adults. It usually presents with an insidious onset and tends to progress over time in adults [5]. Although pediatric MD typically has an abrupt onset, self-limited, and an overall benign course that can spontaneously resolve within 2 to 10 weeks with supportive therapy only [8, 12], uncommon case of non self-limited pediatric MD needing specific treatment was also reported in previous study [11]. Di Nardo et al. reported a non self-limited pediatric MD needing endoscopic mucosal resection for diagnosis which was then successfully treated with octreotide long-acting release (LAR) [11]. MD usually occurs in children younger than 10 years, and boys are affected more often than girls [2]. The mean duration of pediatric MD is less than 6 weeks [10]. The child with MD described in this study was a boy with aged less than 10 years. Studies [10, 13] suggested that pediatric MD patients may present with a variety of nonspecific symptoms. The common symptoms of pediatric MD include abdominal pain, nausea and frequent vomiting, diarrhea, loss of appetite, weight loss, and malnutrition [13]. Peripheral edema due to hypoalbuminemia is also present frequently in pediatric MD patients [8, 10, 13]. In this report, the patient presented with symptoms of abdominal pain and vomiting, and eyelid edema. Radiologic, endoscopic and pathologic findings further supported the diagnosis of MD. Finally, after 2 weeks of supportive therapy, the disease was resolved and the boy was asymptomatic during the follow-up of 6 months.

The exact cause of pediatric MD is still unclear. Previous studies [8, 14–17] have suggested that MD is associated with several gastric infections. The most common infection associated pediatric MD is CMV. It has been reported that around 70% of pediatric MD patients were infected with CMV [8, 18]. Pediatric MD cases with HP infection were also reported in the literature [16]. It was shown that the clinical and biochemical resolution of MD achieved after the eradication therapy for HP infection [14, 15]. Furthermore, *Mycoplasma pneumoniae* infection is reported associated with MD in children [17]. Two weeks before the diagnosis of MD, the patient was infected with *Mycoplasma pneumoniae*, indicating that *Mycoplasma pneumoniae* infection may play a role in the onset of MD.

CD infection is a leading cause of antibiotic-associated and healthcare-associated infective diarrhea, which is determined by the presence of diarrhea and either a detection of toxin producing CD in stool, or findings of pseudomembranous colitis [19]. The prevalence and severity of CD infection in children has been increased in past decades [20]. Antibiotic (metronidazole, vancomycin or fidaxomicin) is the current first line treatment for CDI [19]. Interestingly, the MD case presented here was infected with CD which confirmed by CD toxin test and culture. The CDI may be caused by the large amount of antibiotics using in previous pneumonia episode of the patients. To the best of our knowledge, the association of CD infection with MD has not been reported in the literature. However, the possibility of gastric lesions caused by CD infection-mediated immune disorder can’t be excluded in this reported case. Further studies are needed to investigate the role of CDI on the pathogenesis of MD.

In summary, we described the clinical features of a pediatric case with MD from China in this study. Given the nonspecific symptoms, gastrointestinal endoscopic evaluations with gastric tissue biopsies are required to establish the diagnosis of MD in children with unexplained hypoalbuminemia.

## Data Availability

The data of the current study are available from the corresponding author on reasonable request.

## Abbreviations

CD: *Clostridium difficile*
CMV: Cytomegalovirus
CT: Computed tomography
EBV: Epstein-Barr virus
HP: *Helicobacter pylori*
MD: Ménétrier’s disease
